# The Value of Diaphoresis in Infectious Diseases

**Published:** 1888-09

**Authors:** 


### THE VALUE OF DIAPHORESIS IN INFECTIOUS DISEASE.

A moist skin during the course of infectious and acute diseases has from a very early period in the history of medicine been regarded as a desirable symptom. For the purpose of showing the value of the *rôle* played by the skin in such cases Queiroco, of Genoa, has just published some interesting results of experiments made with sweat upon animals. It is, of course, a point upon which no difference of opinion exists, that the sweat is a potent means by which the materies morbi are eliminated from the organism. The chief cause of the diseases, in the form of toxic elements, ptomaines for example, are dissolved in the sweat, and so extracted from the body. The author collected some sweat from cases of variola, articular rheumatism, typhoid fever, etc., and injected it into various animals. The result of the experiments was that the animals in all cases died. Experimenting, however, with normal sweat taken from healthy men and injecting it into animals, under the same conditions, he found that nothing happened. Thus the value of the old Hippocratic injunction, which makes the administration of plenty of liquids an important element of treatment in acute diseases, is confirmed. It would be interesting to know what, if any, micro-organisms are contained in the sweat of patients suffering from infectious diseases, and whether the fatal results in the animals could be in any way attributed to the effects of microbic invasion through the injections of sweat.—*Medical Press and Circular.*

